# Potassium cations expand the conformation ensemble of *Arabidopsis thaliana* β-amylase2 (BAM2)

**DOI:** 10.17912/micropub.biology.001257

**Published:** 2024-07-22

**Authors:** Abigail Sholes, Roland Asongakap, Sophia Jaconski, Jonathan Monroe, Christopher E. Berndsen

**Affiliations:** 1 Chemistry and Biochemistry, James Madison University; 2 Chemistry and Biochemistry, University of Illinois-Chicago

## Abstract

The process for and regulatory mechanism controlling the synthesis and degradation of the polysaccharide starch are only superficially understood. β-amylases (BAMs) are enzymes that hydrolyze starch into maltose which is further used to drive metabolism and other cellular processes. Most BAMs in plants can function as monomeric enzymes and have hyperbolic kinetics. BAM2 from *Arabidopsis thaliana* is unusual as it forms a homotetramer, displays sigmoidal kinetics, and is stimulated by the presence of potassium cations (K ^+^ ). We used circular dichroism spectroscopy, small-angle X-Ray scattering, and molecular dynamics to investigate the effect of K ^+^ on the structure of BAM2 and found that K ^+^ induces the formation of an active conformation of BAM2 thereby increasing its activity.

**Figure 1. K + expands the conformational ensemble of BAM2:**
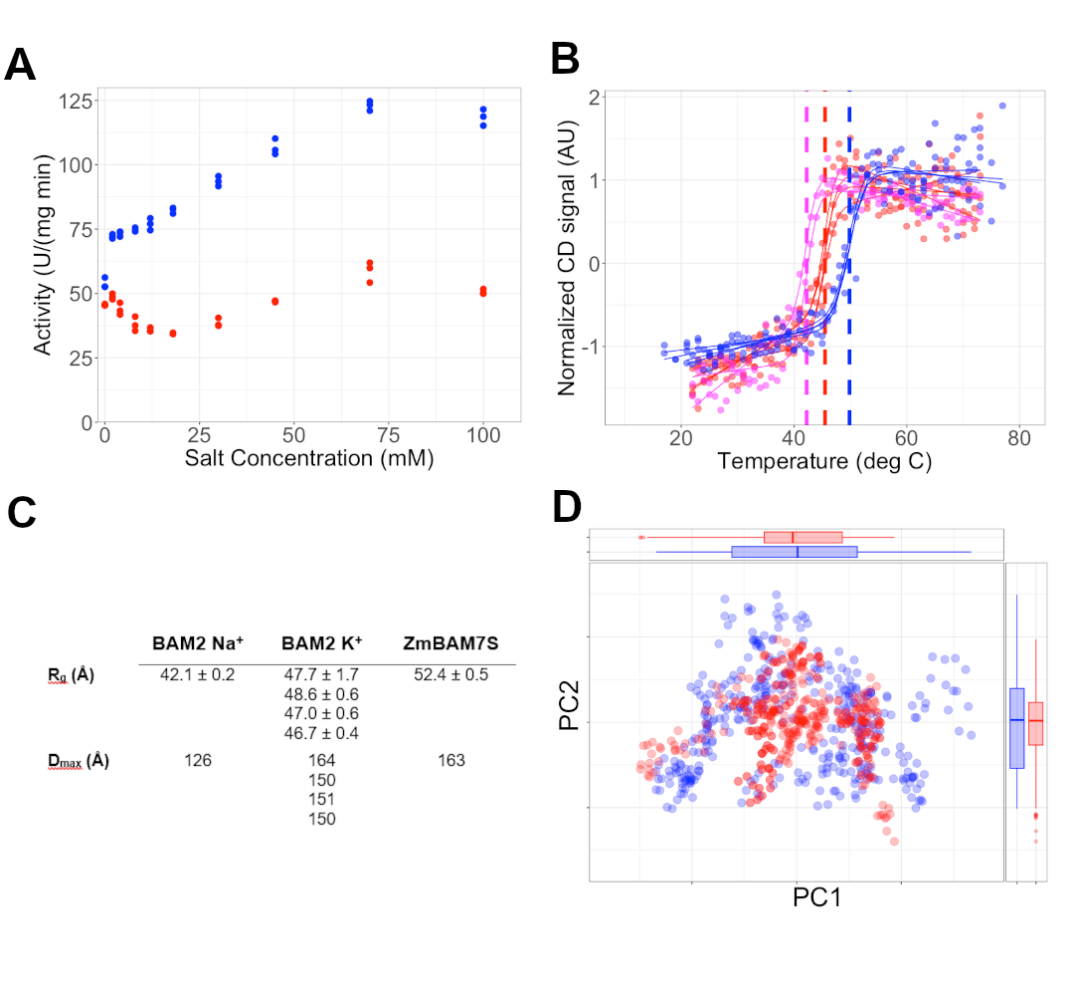
(A) Starch hydrolysis assays of BAM2 with lithium (red) or potassium (blue). Representative data shown are from an assay repeated in triplicate and each measurement is shown. (B) CD melting curves for BAM2 in 0.1 M potassium (blue), 0.1 M lithium (red), and buffer only (magenta). The BAM2 melting temperature in potassium was 49.8 ℃, in lithium 45.5 ℃, and for buffer only 42.2 ℃. The 95% confidence interval for all fits was 0.5 ℃. (C) SAXS statistics of BAM2 in potassium at 0.25 mg/mL, 0.5 mg/mL, 1 mg/mL, and 2 mg/mL compared to BAM2 in Na ^+^ (SASDGY4) and ZmBAM7S (SASDMX5). The raw SAXS data in K+ are available from the SASBDB under codes SASDUZ9, SASDV22, SASDV32, and SASDUY9. (D) Principal component analysis of the BAM2 conformation from simulations in explicit solvent with 0.1 M lithium (red) or 0.1 M potassium (blue). The boxplots show the interquartile range (IQR) from the 25 ^th^ to 75 ^th^ percentile with the whiskers extending 1.5 times the IQR.

## Description

Plants must store energy and carbon for later use. To do so, plants utilize starch that is formed in chloroplasts through the process of photosynthesis. During the day, plants store about half of their photosynthate as starch, which is then utilized almost completely the following night (Smith & Zeeman, 2020) . Starch degradation is also induced in response to biotic and abiotic stress (Thalmann et al., 2016; Thalmann & Santelia, 2017) . In order to convert the starch to available energy, plants utilize a number of enzymes including β-amylases (BAMs) that hydrolyze the starch into maltose. In *Arabidopsis thaliana* , there are nine BAMs, of which six are found in the chloroplast, four of which are catalytically active (Monroe & Storm, 2018) . One of these BAMs, BAM2 (AT4G00490, Uniprot: O65258), is known to have a unique homotetrameric structure and sigmoidal starch hydrolysis kinetics (Chandrasekharan et al., 2020; Monroe et al., 2017, 2018) . In addition, BAM2 activity was enhanced in the presence of potassium, although the mechanistic basis for this enhancement was not clear (Monroe et al., 2017, 2018) .

In previous work, we observed that the presence of Li ^+^ increased BAM2 activity, but not as much as K ^+^ (Monroe et al., 2017) . To determine if Li ^+^ could eventually increase activity to that of K ^+^ or if the effects were more specific to K ^+^ , we performed starch hydrolysis assays at a range of Li ^+^ and K ^+^ concentrations. We found that Li ^+^ showed a minimal increase in activity while K ^+^ showed a clear concentration dependence up to 80 mM when the activity then plateaued with concentration ( Figure 1A ). These data suggest that the effects of K ^+^ are not just due to ionic strength. In addition, levels of K ^+^ in plastids are consistently >100 mM (Demmig and Glimmer., 1983; Schroppel-Meier et al., 1988) . Therefore, it is unlikely that physiological levels of K ^+^ have any regulatory effect on BAM2 activity but likely K ^+^ is required for cellular function.

Next, we aimed to determine the structural role for cations in stimulating BAM2 activity. We first measured the effects of 0.1 M Li ^+^ and K ^+^ on the melting temperature of BAM2 via circular dichroism (CD) spectroscopy and small-angle X-ray scattering. Relative to BAM2 in buffer without additional cations, the melting temperature of BAM2 increased 3 °C in the presence of Li ^+^ and 7 °C in the presence of K ^+^ ( Figure 1B ). The shape of the melting curves was similar across the conditions and the shape of the CD spectra was similar across the temperature range, suggesting that changes in melting temperature were not due to gross changes in conformation. Changes in the voltage measurement at 287 nm during CD melts are used to report the formation of protein aggregates (Du Pont et al., 2016) . We found that for all three conditions, the aggregation temperature was similar to the melting temperature from CD data at 222 nm. Overall, these data suggest that cations enhance the stability of BAM2, with K ^+^ having the greatest effect on BAM2 structure.

BAM2, unlike other BAMs, forms a homotetramer in solution; thus, the increase in melting temperature could be due to a stabilization of the quaternary structure. We addressed this possibility by collecting SAXS data on BAM2 in buffer containing 100 mM K ^+^ and found that the overall shape of the log(I) vs. q-curve matched with data we previously collected on BAM2 in 100 mM Na ^+^ (Chandrasekharan et al., 2020) . These findings suggest that stabilization of the tetrameric form is unlikely to be the basis for the enhancement of BAM2 activity as the protein shape was consistent with a tetramer. We next calculated the R _g_ and D _max_ values which indicate the protein size to determine if K ^+^ had an effect on the apparent proportions of BAM2. Comparison of the protein dimensions between the two conditions shows that the R _g_ and D _max_ values of BAM2 in K ^+^ were larger than those previously determined in Na ^+^ ( Figure 1C ). The BAM2 ortholog from *Zea mays* , BAM7S, which forms a similar homotetramer to Arabidopsis BAM2, had overall larger SAXS dimensions because of extended, and likely dynamic, N-terminal extensions (Ravenburg et al., 2022) . Given that K ^+^ stabilizes BAM2 and increases the melting temperature, the SAXS findings were surprising as they would be consistent with a more dynamic or expanded BAM2 conformation, which is not typically associated with increased stability. Unfortunately, the SAXS data did not allow us to determine where on the protein the changes in conformation were occurring.

To identify where on the structure K ^+^ was acting, we simulated the molecular dynamics of BAM2 in K ^+^ and compared the data to the simulated behavior in Li ^+^ . Consistent with the apparent behavior from the SAXS data, we found that K ^+^ increased the dynamics of BAM2 during simulations. We then compared the ensemble of conformations from all simulations between the two salts and found that the conformational ensemble in K ^+^ is broader than that observed in Li ^+^ ( Figure 1D ). Boxplots in both PC dimensions show a wider interquartile range for simulations in K ^+^ relative to Li ^+^ . These data are consistent with a mechanism whereby K ^+^ lowers the energy barrier for BAM2 to adopt an active configuration, leading to increased catalytic rates. Based on the CD data, this ensemble is apparently more thermostable, leading to the increase in melting temperature. This is further supported by the location of the amino acids with the largest changes in RMSF between the K ^+^ and Li ^+^ simulations. The N- and C-terminal amino acids show the largest deviations from the mean difference. Previous work has shown the N-termini of BAM2 are crucial for tetramer assembly, thus our data suggest that the termini are also involved in the enhancement of activity by potassium (Chandrasekharan et al., 2020) . Overall, the data suggest that K ^+^ enhances protein stability and the structural dynamics of BAM2 leading to increased starch hydrolysis activity. The structural ensemble likely samples the active conformation of BAM2 more frequently permitting a higher catalytic rate. The catalytic rate of TIM barrel proteins is known to depend at least in part on the dynamics of active site loops (Richard, 2019) . Ultimately, these data describe a mechanism of K ^+^ influence on BAM2 activity *in vitro* and demonstrate the importance of considering physiological conditions when characterizing enzyme activity.

## Methods

**Circular Dynamics (CD) Spectroscopy**

For CD measurements, the cuvette contained purified recombinant BAM2 at a concentration of 4 μM in 20 mM MES, pH 6.5, 0.1 mM TCEP, and either no additional salt, 0.1 M KCl or LiCl in a 0.2 cm quartz cuvette. Buffer pH was adjusted with KOH or LiOH as appropriate. Data were collected from 320 nm to 200 nm at 1 nm/sec with 3 accumulations on a Jasco J-1500 CD spectrometer. Spectra were collected at temperatures from 20-75 °C at an interval of 1 °C, with a gradient of 2 °C/min. The sample was equilibrated for 1 minute before spectra were collected at each temperature. Apparent melting temperatures were calculated through a global fit to a one-step equilibrium unfolding model using the Calfitter app (Mazurenko et al., 2018) . Data were exported in .csv formatted and plotted in R using the ggplot2 package.

**Small-Angle X-Ray Scattering (SAXS):**

Recombinant, purified BAM2 was dialyzed into 50 mM HEPES, pH 7, and 0.1 M KCl at 4 °C overnight. Samples at a concentration of 1 mg/mL in a plate were shipped overnight on dry ice to the SIBYLS beamline at the Advanced Light Source (Dyer et al., 2014) . Prior to data collection, the plate was spun at 3700 rev min ^-1^ for 10 min. Samples were held at 10 ℃ during collection. The exposure was 10 s, with frames collected every 0.3 s for a total of 30 frames per sample. The detector was 2 m from the sample, and the beam energy was 11 keV. Matching buffer exposures were collected before and after samples to ensure there was no difference in the scattering owing to contamination of the sample cell. This setup results in scattering vectors, q, ranging from 0.013 Å ^-1^ to 0.5 Å ^-1^ , where the scattering vector is defined as q = 4πsinθ/λ and 2θ is the measured scattering angle. Radially averaged data were processed in SCATTER (ver 4.0d) and RAW (ver. 2.1.1) to remove the scattering of the sample solvent and identify peak scattering frames (Hopkins, 2024) . RAW was used to calculate the dimensions of the molecule, the molecular weight, and the pair-distance distribution function (PDDF). SASBDB codes are SASDUZ9, SASDV22, SASDV32, and SASDUY9 and the raw data are available at SASBDB (Kikhney et al., 2020) .

**Molecular Dynamics**

Models of the BAM2 tetramer in 0.1 M K ^+^ or Li ^+^ were made in CHARMM-GUI and simulations were run in OpenMM with an AMBER19ffsb forcefield (Lee et al., 2016; Eastman et al., 2017) . Data were analyzed using Bio3D (Grant et al., 2021) .

**Activity assays**

BAM2 activity with soluble starch as the substrate was measured using the procedure described by Monroe, et al. with the exception of monovalent cation concentrations (Monroe et al., 2017) .

## Extended Data

Description: The .dat and .out files for the SAXS data. Concentration is indicated in the filename.. Resource Type: Dataset. DOI: 10.22002/er2te-58p41
